# Setting up and sustaining blood and marrow transplant services for children in middle-income economies: an experience-driven position paper on behalf of the EBMT PDWP

**DOI:** 10.1038/s41409-020-0983-5

**Published:** 2020-09-07

**Authors:** Lawrence Faulkner, Marta Verna, Attilio Rovelli, Rajat Kumar Agarwal, Rakesh Dhanya, Lalith Parmar, Amit Sedai, Ankita Kumari, Stalin Ramprakash, C. P. Raghuram, Pallavi Mehta, Sandeep Elizabeth, Sadaf Khalid, Aliya Batool, Sarah Khan Ghilani, Itrat Fatima, Tatheer Zara, Priya Marwah, Rajpreet Soni, Deepa Trivedi, Valentino Conter, Marta Canesi, Dosti Othman, Vian Faeq, Katharina Kleinschmidt, Akif Yesillipek, Catherine G. Lam, Scott C. Howard, Selim Corbacioglu, the Pediatric Diseases Working Party of the European Blood and Marrow Transplantation Group, Abdulah Al Jefri, Alice Bertania, Jochen Büchner, Andrè Willasch, Brenda Gibson, Tayfun Güngür, Marianne Ifversen, Roland Meisel, Ingo Müller, Kim Vetteranta, Paul Veys, Jacek Wachowiak

**Affiliations:** 1 Cure2Children Foundation, Florence, Italy; 2 Sankalp India Foundation, Bengaluru, India; 3 MBBM Foundation, Pediatric Department, University of Milano-Bicocca, Monza, Italy; 4 People Tree Hospitals, Bengaluru, India; 5 Dr Akbar Niazi Teaching Hospital, Islamabad, Pakistan; 6 Children’s Hospital Pakistan Institute of Medical Sciences, Islamabad, Pakistan; 7 South East Asia Institute for Thalassemia, Jaipur, India; 8 CIMS Hospital, Ahmedabad, India; 9 Hiwa Hospital, Sulaymaniyah, Kurdistan Region Iraq; 10 Department of Pediatric Hematology, Oncology and Stem Cell Transplantation University of Regensburg, Regensburg, Germany; 11 Medicalpark Antalya Hospital Pediatric Stem Cell Transplantation Unit, Antalya, Turkey; 12 Department of Global Pediatric Medicine, St Jude Children’s Research Hospital, Memphis, TN USA; 13 University of Tennessee Health Science Center, Memphis, TN USA; 14 King Faisal Specialist Hospital, Riyadh, Saudi Arabia; 15 Lucile Salter Packard Children’s Hospital, Stanford, CA USA; 16 Oslo University Hospital, Oslo, Norway; 17 Goethe University, Frankfurt, Germany; 18 University of Glasgow, Glasgow, UK; 19 University Chilren’s Hospital Zurich, Zurich, Switzerland; 20 Copenhagen University Hospital, Copenhagen, Denmark; 21 Heinrich-Heine-University, Düsseldorf, Germany; 22 University Medical Center of Hamburg-Eppendorf, Hamburg, Germany; 23 Children’s Hospital, University of Helsinki, Helsinki, Finland; 24 Great Ormond Street Hospital NHS Trust, London, UK; 25 University of Medical Sciences, Poznan, Poland

**Keywords:** Paediatrics, Health services

## Abstract

Severe blood disorders and cancer are the leading cause of death and disability from noncommunicable diseases in the global pediatric population and a major financial burden. The most frequent of these conditions, namely sickle cell disease and severe thalassemia, are highly curable by blood or bone marrow transplantation (BMT) which can restore a normal health-related quality of life and be cost-effective. This position paper summarizes critical issues in extending global access to BMT based on ground experience in the start-up of several BMT units in middle-income countries (MICs) across South-East Asia and the Middle East where close to 700 allogeneic BMTs have been performed over a 10-year period. Basic requirements in terms of support systems, equipment, and consumables are summarized keeping in mind WHO’s model essential lists and recommendations. BMT unit setup and maintenance costs are summarized as well as those per transplant. Low-risk BMT is feasible and safe in MICs with outcomes comparable to high-income countries but at a fraction of the cost. This report might be of assistance to health care institutions in MICs interested in developing hematopoietic stem cell transplantation services and strengthening context appropriate tertiary care and higher medical education.

## Introduction

The spectrum of bone marrow transplant (BMT) indications in the pediatric age group is wide; in particular, hemoglobinopathies are the most common life-threatening noncommunicable diseases in children [1]. Transplantation can cure over 85% of low-risk children with sickle cell disease (SCD) and severe thalassemias (ST) in lower-middle- and upper-middle-income countries (MICs) [2], improves long-term health-related quality of life [3], and is cost-effective [4]. BMT is often also the only life-saving option for high-risk hematological malignancies, marrow failure syndromes, and other severe disorders.

In many MICs BMT units are lacking, insufficient to meet the demand, or financially inaccessible to most patients [5]. Not infrequently, children requiring BMT and their families have to leave their home country, increasing hardship and perpetuating the hemorrhage of financial and human resources to more affluent countries.

Recently, the Worldwide Network for Blood & Marrow Transplantation published general recommendations for establishing BMT programs in countries with limited resources, focusing on adult transplantation [6, 7]. There are, however, substantial differences between pediatric and adult transplantation, particularly in MICs, including the spectrum of indications (primarily nonmalignant diseases in children as opposed to hematological malignancies in adults), type of BMT (largely allogeneic in children), and posttransplant course with younger patients having less GVHD as well as other complications.

This paper summarizes recommendations based on real-world experiences and outcomes from close to 700 allogeneic BMTs performed in several newly setup BMT services across South-East Asia and Middle East from 2009 to 2020.

## Getting started

Engaging local professionals and other stakeholders with the proper commitment is the first and foremost step. Financial issues are important, but probably even more relevant is to build professional relationships based on shared principles and vision, create consensus, and a patient referral base. In most MICs, where drugs and consumables require importation or where BMT-specific tests need international outsourcing, local governments may be pivotal in facilitating those transfers and to avoid cost escalations related to custom charges and intermediaries. Whenever possible, local health authorities should be involved from the start to increase long-term sustainability of the project. However, government officials may move on to other duties and be replaced by others with different agendas, so that nongovernmental resources should also be relied upon. There should be a close association between referring centers and BMT services in order to optimize transplant outcomes. This is particularly true for hemoglobinopathies, where patients’ condition at transplant is a major determinant of outcome [8]. While developing BMT services, it is also important to consider creating or aligning with programs for supportive care needs, such as hydroxyurea treatment and early diagnosis for SCD, or iron chelation and safe transfusion for ST. The same holds true for other indications such as high-risk leukemias where the implementation of proper diagnostic and therapeutic standards, as well as accurate remission assessment, is critical [9]. Particularly in low-resource settings, to minimize the likelihood of adding financial catastrophe to emotionally drained families, BMT should be offered when the probability of cure is substantial.

Offering free or subsidized HLA typing (high resolution where appropriate, particularly in communities with high consanguinity rates or close ethnicity [10]), may help to engage families in addition to building a queue of appropriate BMT candidates. HLA typing can be reliably outsourced internationally via buccal swab DNA collection kits. Setting up local HLA laboratories is no longer an absolute necessity provided outsourcing turnaround times are acceptable for the spectrum of BMT indications being considered. However, local independent confirmatory typing remains desirable.

Blood banks should be involved from early setup stages and local blood supply quality assurance should be assessed on a high priority basis. While leukodepletion may not be essential, blood product irradiation is, since the former might not be sufficient to prevent transfusion-associated GVHD [11]. Local factors, such as frequency of sickle cell carriers among blood donors and transfusion-transmitted malaria, should be kept in mind.

To facilitate close communication, monitoring, and evaluation of programmatic as well as clinical outcomes, an online password-protected dedicated information technology platform is very important [12]. In fact, the overall BMT development project will necessarily have to rely on a mixture of on-site and online interactions in both the preparation/training phase as well as during the implementation phase.

## Autologous vs. allogeneic transplantation at start-up

Topically pediatric allogeneic transplant programs have started from general hematology–oncology services moving onto autologous transplantation and eventually to allotransplants. However, autologous transplantation in children is technologically more demanding than allogeneic BMT, as it relies on leukapheresis in small children, CD34^+^ cell enumeration by flow cytometry, and cryopreservation. This is quite different from adult autologous transplantation where the indication is significantly more frequent than in children, and where regimens employed may be less intensive and not necessarily require cryopreservation. The preferred source of transplantable hematopoietic stem cells in the pediatric setting is generally bone marrow, particularly for nonmalignant conditions [13], which is easy to collect, relies on simple nucleated cell count, and does not require cryopreservation. The need for an autologous back up is questionable [14]. Considering all these peculiarities, it seems reasonable in the pediatric setting to start directly from allogeneic transplant before moving to autologous, which generally has more limited indications and success rates.

To successfully launch a low-risk allogeneic BMT program as a first step of a multidimensional BMT program, it is essential to establish a structured cooperation program involving expert BMT physicians and nurses with committed local personnel, and a highly manageable target patient: BMT for ST or SCD is an appealing starting point in many settings as it involves only a few drugs, such as fludarabine, antithymocyte globulin, busulfan, cyclophosphamide, cyclosporine, and methotrexate, and a single repetitive treatment protocol, which is thus amenable to focused training [2]. The use of information technology platforms generating patient-specific treatment regimens designed according to good clinical practices is important to provide clear and legible orders, and minimize prescription errors in a context of relatively complex therapy with potentially toxic drugs [12]. Patients with hemoglobinopathies also allow ample time to optimize pre-BMT clinical conditions and, given the lack of prior exposure to chemotherapy, generally tolerate conditioning quite well. Children with malignant diseases usually have received intensive chemotherapy prior to transplant, are at higher risk of severe complications, and may require more complex transplant facilities.

Families with a child suffering from a malignancy or a hemoglobinopathy are typically aware of the physical, psychological, and financial burden of disease as well as treatment. However, treatment abandonment may still occur, mostly related to financial or cultural issues. This aspect needs to be considered, with appropriate financial and psychosocial counseling and support integrated into successful programs.

## BMT unit design and infection control

Technical and professional aspects such as start-up BMT unit location, size, design, infection control procedures, and, most importantly, roles and responsibilities, need to be addressed. The design of the unit may vary considerably from country to country according to available structures, funding, local epidemiology in terms of case load and case mix, and BMT activity prospects. It might be advisable to start with a relatively small four-bedded BMT unit, allowing sizable BMT activity (at least in low-risk patients) of 20–30 BMTs/year, while building a core team (see Table 1). BMT unit design should be simple and equipment reduced to a minimum. Setting up such BMT units for low-risk patients within an existing hospital facility should not cost more than US$100,000 including all basic equipment (see Table 2), furniture and appliances included [15]. Keeping infusion pumps and monitors outside BMT rooms and connected to patients through small wall or glass openings has been adopted in some centers to minimize nurse trafficking into patient rooms, thus decreasing the risk of transmitting hospital-acquired drug-resistant organisms and increasing work efficiency. Private en suite bathrooms might not be generally required, as young children may prefer to use bedside pans; parents may use centralized bathroom facilities within the BMT unit, which would also ideally host a washing machine and dryer. Keeping BMT rooms dry and easy to clean and avoiding wastewater/plumbing is very important in terms of infection control [16, 17].

**Table 1:** Core team.

Role	Workforce
Physicians^a^	2 (1 program director and 1 BMT unit physician)
Nurses	10 (1 nursing coordinator and 9 unit nurses)
General manager/secretary	1
Psychologist/psychosocial coordinator	1
Housekeeping staff	2

Suggested minimum core team for a four-bedded BMT unit performing 20–30 BMTs per year.

^a^Depending on qualifications and experience of BMT physicians, supporting BMT experts on-site and online may be required.

**Table 2:** Equipment (a): essential equipment for a four-bedded BMT unit; (b) essential imaging. All except MRI maybe required on an emergency basis and should ideally be in-house. MRI maybe outsourced locally; (c) essential additional consumables for BMT harvest and central venous access.

	Units
a: *Equipment*	
**Blood Cold Chain Box (insulated cool box)**	2
**Blood pressure apparatus**	4
**Infusion pumps**	4
**Laminar flow sterile hood**	1
**Medication Trolley**	2
**Nebulizer**	1
**Oxygen cylinder**	2
**Patient monitor (including O2 saturation)**	4
**Portable Crash Box/resuscitation trolley**	1
**Portable Pulse Oximeter**	2
**Refrigerator +4 °C with temperature monitoring and alarm**	2
**Syringe pumps**	4
Tube sealer	1
**Weighing scales (regular and for infants)**	4
**Wheel chair**	1
**X-ray viewer**	1
b: *Imaging*	
**CT Scan**	
**Electrocardiogram (ECG)**	
**MRI**	
**Ultrasound (including Doppler) and echocardiogram**	
**X-ray**	
c: *Marrow collection and central venous access*	
Blood bag spike with **Luer lock connection**	
Blood transfusion set (200 µ mesh)	
Disposable **Bone Marrow Needles**	
Standard blood bank bags	
**Transparent film dressings**	
Tunneled **central lines**	

Equipment included in WHO Priority Medical Devices for cancer and/or children are noted in bold.

There are no well-designed prospective studies establishing the requirement for complex air control systems [18]. International guidelines do not call for high-efficiency particulate air filtration and positive pressure BMT rooms for low-risk transplants [19]. Moreover, repair and maintenance of complex centralized air control systems may not be always easy in MICs, and technical problems may paralyze transplant services for prolonged periods. Split air conditioning with frequent cleaning and thorough maintenance will be required in most MICs but not necessarily complex air control systems, at least for low-risk transplants. For higher risk patients, portable air purification devices might be preferable. In the experience of the Cure2Children Foundation in several BMT units across the Indian subcontinent lacking air control systems but implementing proper hand hygiene practices and basic precautions in several hundred matched related BMTs for low-risk thalassemia patients, outcomes were at par with international standards with no evidence of increased air-borne opportunistic infections, e.g., aspergillosis [2].

Another issue to be kept in mind is the rise of antibiotic-resistant organisms, where the patient-to-patient spread has little to do with air control but rather with nursing care and judicious use of antibiotics. Strict handwashing of all personnel and visitors needs to be enforced and BMT units cleaned daily by dedicated personnel with clear and well-defined procedures. These precautions, together with skilled central venous line care, might be enough to control most severe infectious complications.

## Support services

BMT will require several ancillary services starting from pediatric anesthesia and intensive care to operating room, radiology department, radiotherapy for total body irradiation (primarily required for acute lymphoblastic leukemia) and for blood product irradiation if a blood irradiator is not available. A number of subspecialists as consultants will also be required, from infectious diseases to cardiology, nephrology, gastro-enterology, and nutrition, as specified in international accreditation guidelines [19]. Ideally, these services should be in-house, but this is not an absolute requirement provided appropriate referral and transfer procedures are in place. For example, it is safer and more cost-effective to have a good pediatric ICU within a reasonable distance from the BMT unit rather than one without the appropriate expertise in-house.

## Transplant costs

The World Bank defines lower-middle-income economies according to gross national income per capita between US$996 and US$3.895 [20]. In these regions, universal health coverage is generally not available and most medical expenses are paid out-of-pocket, so that cost variations of a few thousand US$ can constitute more than a year’s average salary and have a major impact on transplant accessibility. Thus, eliminating every expense not justified by hard evidence of positive impact on relevant outcomes is paramount to maximize accessibility and minimize cost-related deaths. In the Sankalp-C2C Network experience, a matched sibling BMT for a low-risk child with severe thalassemia can be performed for an average of <US$15,000 with all medical costs included (Table 3). This cost containment is primarily related to reduced hospitalization charges because of simplified BMT units with low construction and maintenance costs, autonomous financial administration of BMT units, and negotiated network rates for drug procurement and diagnostic investigations. The costs quoted in Table 3 are based on real-world programmatic costs at 1 year from BMT for children with thalassemia with low-risk features, in good conditions pre-BMT, receiving a matched related graft and aggressive GVHD prophylaxis. Moreover, in India nurses’ salaries as well as the cost of drugs, investigations, and blood products are often less than in most other MICs, so that these cost figures may need to be adjusted for local factors. Possible, probable, or proven invasive fungal infections, the treatment of which may substantially impact on final costs because of increased hospital stay and costly drug utilization, occurred in only 2% of transplants, in spite of lack of strict air control systems [21, 22]. Moreover, the prevention of GVHD, which can substantially impact on overall outcomes and costs, was pursued by avoidance of peripheral blood stem cell collections [23], use of double drug prophylaxis, and G-CSF primed bone marrow [24]. Similar allogeneic BMT costs, i.e. US$<20,000 per transplanted patient, have also been reported from other institutions in India [25]. Slightly increased costs are estimated in other areas such as Africa, Central and South America. An important consideration to be kept in mind when comparing BMT prices is to evaluate the extent of coverage provided.

**Table 3:** BMT costs.

	SEAIT	PTH	CIMS
	(4 bed unit)	(4 bed unit)	(4 bed unit)
Minimum	$7945	$6230	$9822
25% Percentile	$9439	$9422	$11,346
Median	**$9943**	**$10,741**	**$11,921**
75% Percentile	$10,240	$12,592	$13,087
Maximum	$29,813	$40,289	$24,102

Range in costs per patient in US$ across three BMT units in India. All costs are up to 1 year post BMT.

*SEAIT* South-East Asia Institute for Thalassemia—Jaipur, *PTH* People Tree Hospital—Bengaluru, *CIMS* CIMS Hospital—Ahmedabad.

As soon as the center achieves enough experience in low-risk BMT the question is how to meet the needs of those patients lacking a matched family donor. The access to alternative stem cell sources such as unrelated donors or cord blood units in MICs is often impossible due to financial, organizational, or ethical constraints. Partially matched related (haploidentical) transplantation with posttransplant cyclophosphamide might thus be a cost-effective alternative [26, 27].

## Training strategy, personnel, and core competencies

Investing in trained nurses and physicians, alongside proper supervisory and clinical support mechanisms and continuing quality improvement programs aiming at eligibility for international accreditation should be top priorities. Nursing personnel’s desirable qualifications include experience with the management of sick children or newborns and handling of central venous lines.

Our experience supports a training model by which expert BMT physicians and nurses travel to new BMT unit sites and supervise hands-on training of the local BMT team. This appears to be more effective and less expensive than having a few local physicians or nurses travel to established transplant centers. Several challenges that need to be addressed in the setup phase need close local supervision and a strong problem-solving attitude. On-site training should start when all basic requirements are in place and at least three appropriate BMT candidates have been identified and their families have consented to transplantation. A team of professionals composed of nurses and doctors with BMT experience rotates during the first BMTs covering from a few days before the start of the first patient’s preparative regime to at least day +30 of the last patient, thus staying on site for a minimum of ~2 months. International staff, even if coming from different hospitals, should prepare in advance to align approaches and educational key messages. This initial on-site training should be followed by daily interaction via a web-based electronic medical record system and teleconferencing as needed. At least one rotating “shadow” experienced BMT physician and nurse should be available online 24 h a day for 7 days a week for several months until the core local team is consolidated and self-confident. A comprehensive contextappropriate BMT manual including all major clinical practice guidelines continuously updated in collaboration with local medical and nursing staff should be available.

Particularly in the start-up phase, experienced nurses are a critical component of BMT programs. In fact, many management decisions can be discussed with relatively inexperienced local physicians by online tools but there is no web-based substitute for proper nursing care. In many MICs, nursing shortage and turnover is likely to be a problem. There are several reasons for this, ranging from cultural issues, poor salary, and insufficient professional motivation. BMT units should create a welcoming environment with a minimum of five to six nurses covering a shift and a nurse to bed ratio of 1:2 [28]. Professional development plans should be in place, possibly leading to a career as an advanced practice provider with proper salary adjustments.

## Drugs, consumables, and laboratory tests

Costs of supplies, medicines, and laboratory tests can vary widely, and, as a general principle, the least expensive but established supplies, medicines, and investigations should be employed. Judicious use of antibiotics will be very important to minimize drug resistance and fungal infections. In terms of BMT-specific tests, the ones that are most important are cyclosporine or tacrolimus blood levels, and CMV DNA copy number by real-time PCR. Donor chimerism is also required, but probably less critical for nonmalignant indications. See Tables 4 and 5 for a list of suggested essential medicines, supplies, and investigations for pediatric BMT. Notably, we have performed a comparison of these recommended essential needs for BMT with the most recent World Health Organization (WHO) model lists of essential medicines [29] and in-vitro diagnostics [30] for priority health care needs globally, as well as priority medical devices for cancer [31], and for child health [32]. Reassuringly, the majority of our recommendations for pediatric BMT are already included within WHO’s model essential lists and recommendations used by governments around the world to prioritize resource allocation for various indications, not specifically oriented to BMT—supporting the case that specialized BMT programs can be synergistic with other priority health services at a population level. To address some of the key remaining gaps, this group plans to coordinate comprehensive proposals to WHO to support the inclusion of BMT-related needs in upcoming revisions of the essential lists.

**Table 4:** Essential medicines and products for most pediatric BMT indications.

***Acyclovir***	DMSO (dimethyl sulfoxide)	***Levofloxacin***	Pheniramine maleate
***Albendazole***	***Dexamethasone***	***Meropenem***	***Phenytoin***
Albumin	***Epinephrine***	***Lidocaine***	Phosphorus
Aluminum and magnesium hydroxide	***Etoposide***	***Lorazepam***	***Piperacillin/tazobactam***
***Amikacin***	***Dextrose (glucose)***	**Melphalan**	***Potassium chloride***
**Amlodipine**	***Filgrastim***	Magnesium	Potassium phosphates
***Amoxicillin and clavulanic acid***	**Fludarabine**	***Methotrexate***	***Prednisolone***
***Amphotericin B***	***Enalapril***	***Mesna***	Probiotic
Antithymocyte globulin (ATG) rabbit	**Fentanyl**	***Methylprednisolone***	***Ringer lactate (RL) (sodium lactate)***
***Azithromycin***	***Folic acid***	***Metronidazole***	***Salbutamol***
***Budesonide***	***Furosemide***	***Morphine***	Sodium bicarbonate
Busulfan	Ganciclovir	Multivitamin	***Spironolactone***
***Chlorhexidine***	***Heparin sodium***	Mycophenolate	***Sulfamethoxazole*** ***+*** ***trimethoprim***
***Calcium gluconate***	***Cyclophosphamide***	Levosalbutamol	Tacrolimus
***Ceftriaxone***	***Hydrocortisone***	***Normal saline (sodium chloride)***	Thiotepa
***Cefixime***	***Folinic acid (calcium folinate)***	***Oral rehydration solution***	Tramadol
Chloral hydrate	***Hydroxyurea (hydroxycarbamide)***	***Ondansetron***	**Tranexamic acid**
Chlorphenamine or chlorpheniramine	***Intravenous immunoglobulin (IVIG)***	Pantoprazole	***Valganciclovir***
**Clotrimazole**	Iron-multivitamin combination	***Paracetamol***	***Vancomycin***
Colistimethate	Lansoprazole	Paraffin emollient cream	Vitamin K
***Cyclosporin***	***Lactulose***	***Penicillin***	***Voriconazole***
***Cytarabine***	Levetiracetam	Phenazopyridine	Zinc-multivitamin combination

Bolded medicines are included in the 2019 WHO Essential Medicines List (for adults and children age 13 and above); bolded and italicized medicines are also included in the 2019 WHO Essential Medicines List for Children (up to age 12).

**Table 5:** Essential laboratory tests for matched related BMT.

CMV PCR (quantitative)	**D-dimer**	Ionized calcium	Urine sodium
**Blood/urine culture**	**Cultures and sensitivity body fluids**	**Iron**	**SGPT (ALT)**
**Albumin**	**CRP**	Isohemagglutinins titers	**Sodium**
CMV PCR (qualitative)	HHV6	**LDH**	**Stool culture**
**Alkaline phosphatase**	**Hemoglobin electrophoresis (HPLC)**	**Lipase**	**Stool ova and parasites**
**Bilirubin**	**Direct Coombs test**	**Magnesium**	**Syphilis**
**Blood cross match**	Donor chimerism	Parvovirus B19 DNA PCR	T3,T4, **TSH**
**Bone marrow aspiration studies**	**Ferritin**	**Peripheral smear**	Tacrolimus levels
**Calcium**	Cyclosporine level	Phosphorus	**Total Protein**
**Chloride**	**Fibrinogen**	**Potassium**	**Urea (blood urea nitrogen, BUN)**
Clostridium difficile toxin	**Glucose**	**Procalcitonin**	Varicella IgG
CMV serology	**HBsAg**	**PT-PTT**	**Venous blood gases**
**Complete blood count**	**HIV I and II**	Reticulocyte count	
**Creatinine**	**Immunoglobulin G**	**Urine B-HCG (pregnancy test)**	

Tests included in the WHO 2019 Second Model List of Essential In-Vitro Diagnostics are bolded.

As the BMT unit is being developed it might be important to be aware about international accreditation standards [19] and the relevance of scientific publications to enable an effective investment and advocacy case for pediatric BMTs to be presented to governments and funders.

## How could the EBMT PDWP outreach subcommittee and other scientific organizations help?

There are several ways international scientific bodies may provide support to BMT centers in MICs, including: online and on-site training and consultation, patient referral, training programs for physicians and nurses, collaboration and guidance for the design of clinical studies, biostatistics, and quality assurance programs.

## Summary

Within structured collaboration programs focused on common severe disorders which are highly curable by BMT, it is possible to develop single or networked BMT units in MICs to perform safe and effective BMT at a relatively low cost. This can be achieved with on-site involvement of BMT professionals from established centers followed by online interactions and support. The cure of severe hemoglobinopathies offers a unique opportunity to achieve high success rates from the very beginning of transplant activity and therefore might be the ideal platform for future development of BMT programs.
